# Hydrophobic and stretchable Ag nanowire network electrode passivated by a sputtered PTFE layer for self-cleaning transparent thin film heaters†

**DOI:** 10.1039/c8ra00880a

**Published:** 2018-05-22

**Authors:** Sang-Mok Lee, Sung Hyun Kim, Jae Heung Lee, Sang-Jin Lee, Han-Ki Kim

**Affiliations:** Kyung Hee University, Department of Advanced Materials Engineering for Information and Electronics 1 Seocheon-dong Yongin-si Gyeonggi-do 446-701 Republic of Korea; Chemical Materials Solutions Center, Korea Research Institute of Chemical Technology 141 Gajeongro, Yuseong Daejeon 305-600 Republic of Korea; School of Advanced Materials Science & Engineering, Sungkyunkwan University 2066 Seobu-ro, Jangan-gu Suwon Gyeonggi-do 440-746 Republic of Korea hankikim@skku.edu

## Abstract

We demonstrated hydrophobic, flexible/stretchable, and transparent electrodes made up of Ag nanowire (NW) networks passivated by a sputtered polytetrafluoroethylene (PTFE) layer to produce self-cleaning transparent thin film heaters (TFHs). Using carbon nanotubes and a PTFE mixed conducting target, we successfully sputtered a transparent PTFE layer on the Ag NW network using mid-frequency magnetron sputtering. The hydrophobic surface of the PTFE/Ag NW electrodes led to water-repelling and self-cleaning transparent Ag NW electrodes, which are beneficial for transparent TFH-based smart windows. Furthermore, hydrophobic PTFE/Ag NW electrodes coated on polyethylene terephthalate (PET) and polyurethane (PU) substrates showed outstanding flexibility and stretchability, respectively, due to the capping effect of the PTFE layer. Based on outer/inner bending and stretching test results, we demonstrated the superior mechanical properties of the PTFE/Ag NW electrode compared to a bare Ag NW electrode. Finally, we investigated the feasibility of the PTFE/Ag NW film coated on a PU substrate as a transparent and stretchable electrode for stretchable and self-cleaning transparent TFHs. The effective heat generation of the stretchable PTFE/Ag NW electrode indicates the potential for energy-efficient multi-functional PTFE/Ag NW-based TFHs attached to automobile windows.

## Abstract 2

We demonstrated hydrophobic, flexible/stretchable, and transparent electrodes made up of Ag nanowire networks passivated by a sputtered polytetrafluoroethylene layer to produce self-cleaning transparent film heaters.

## Graphical abstract

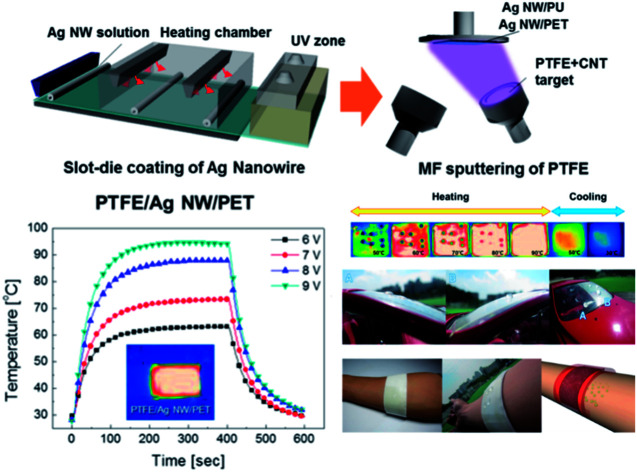

## Introduction

Transparent thin film heaters (TFHs) have attracted much interest for use in defogging/de-icing windows and as heating sources for automobiles, displays, sensors, reaction cells, microchips, and transparent vinyl-based greenhouses.^1–3^ In particular, the demand for flexible or stretchable TFHs that can be attached to curved windows or the human body has rapidly increased due to advances in smart windows and wearable electronics.^4,5^ Among the several components which comprise transparent and stretchable TFHs, high-performance transparent electrodes with low resistivity, high transparency, and outstanding mechanical flexibility/stretchability are very important because the power injection, heat generation properties, heating uniformity, and transparency of TFHs are critically affected by the electrical, optical, and mechanical properties of the electrodes. Particularly for TFHs that are to be attached to building windows, automotive windows, or the human body, hydrophobicity and self-cleaning surface properties are also required as well as mechanical stretchability. Therefore, the development of water-repelling hydrophobic and stretchable transparent electrodes is very important in order to realize self-cleaning and stretchable TFHs. Until now, most transparent TFHs have employed sputtered ITO films as a transparent electrode due to their low resistivity, high transparency, large area scalability, and mass productibility.^6^ However, the easy crack formation, hydrophilic surface, and expensive indium element in ITO films are critical problems with conventional ITO electrodes for the production of self-cleaning, flexible/stretchable, and cost-effective TFHs.^7–9^ For this reason, several alternatives have been reported as electrodes for transparent TFHs, including carbon-based electrodes (carbon nanotubes, graphene, reduced graphene), conducting polymers, nanostructured metal electrodes (grid, nanowire, network), and hybrid electrodes.^10–15^ Among several transparent electrodes, printable Ag nanowire (NW) networks in particular exhibited low sheet resistance, high transmittance, and outstanding mechanical properties.^16–18^ However, most transparent Ag NW electrodes do not have sufficiently superhydrophobic surface properties to repel water and maintain a clean surface on the transparent TFHs. Although chemical treatment and plasma surface treatment have been employed to make the electrodes superhydrophobic, the surface properties of the transparent electrodes ultimately changed back to their original state with increasing time.^19^ Therefore, to realize self-cleaning and flexible/stretchable TFHs, the development of low resistance and flexible/stretchable transparent electrodes with superhydrophobic surface properties is imperative. In spite of their importance, there are no reports on hydrophobic and flexible/stretchable Ag NW network electrodes for self-cleaning and transparent TFHs.

In this study, we report highly flexible/stretchable, transparent, and superhydrophobic Ag NW network electrodes passivated by polytetrafluoroethylene (PTFE) using a mid-frequency (MF) magnetron sputtering to realize self-cleaning and transparent TFHs. We also compared the properties of bare Ag NW electrodes with those of the PTFE/Ag NW electrodes to show the effect of the MF-sputtered hydrophobic PTFE passivation layer. In addition, the mechanical flexibility and stretchability of the PTFE/Ag NW network electrodes on flexible PET and stretchable PU substrates were comprehensively investigated to determine their suitability as transparent electrodes for flexible/stretchable TFHs with hydrophobic surfaces. The results clearly showed the potential of the PTFE/Ag NW electrode for use in a wide range of applications, such as self-cleaning de-icing and defrosting windows for automobiles, self-cleaning outdoor solar cell devices, self-cleaning greenhouses, skin/wearable electronics, and waterproofing bio-patches.

## Experimental

### Fabrication of Ag NW electrodes on flexible PET and stretchable PU substrates

100 mm wide Ag NW network films were prepared on a PET substrate and PU substrate at room temperature using a specially-designed pilot-scale roll-to-roll (RTR) slot-die coating system (DKT 2015-R1-SHU500). The diameter of Ag NW network between 15 and 25 μm and the length is between 15 and 25 μm. The surface Ag NWs was covered with polyvinylpyrrolidone (PVP). The RTR slot-die coating system consisted of ink tanks, a slot-die coating zone, a substrate heating zone, and a UV treatment zone. Using the RTR slot die coating system, an Ag NW layer was uniformly coated on PET through the slot-die head and then the films were moved to the heating zone by means of unwinding and rewinding. The Ag NW network was also coated on the A4-size PU substrate, which was attached to the PET substrate roll under the same slot-die coating conditions.

### MF sputtering of hydrophobic PTFE passivation layer

The PTFE passivation layer was sputtered onto the Ag NW-coated PET and PU substrates using an MF magnetron sputtering system at room temperature using 4-inch PTFE (A7-J, Dupont Mitsui, 95 wt%) and CNT (HANOS CM-280, Hanwha Chemical, 5 wt%) mixed conductive target. For the MF magnetron sputtering of the PTFE layers, we used a dual cathode power source (PE II 5 kW power supply, Advanced Energy). The PTFE layer was directly sputtered onto Ag NW/PET and Ag NW/PU samples at a constant MF power of 300 W (power density 3.69 W cm^−2^) under an Ar flow rate of 50 sccm and a working pressure of 7 mtorr. The distance between the PTFE target and the substrate was held at 240 mm. The thickness of the PTFE passivation layer was controlled by the sputtering time.

### Analysis of PTFE/Ag NW/PET and PTFE/Ag NW/PU

The electrical and optical properties of PTFE/Ag NW prepared on PET and PU substrates were examined by Hall measurement (HL5500PC, Accent Optical Technology) and a UV/visible spectrometer (UV 540, Unicam) as a function of the thickness of the PTFE layer. The microstructures and interface of the optimized PTFE layers on the Ag NWs were examined using high-resolution electron microscopy (HREM). Fast Fourier transform (FFT) images were obtained from a cross-sectional HREM specimen prepared using focused ion beam (FIB) milling. The mechanical properties of the PTFE/Ag NW electrodes were evaluated using a specially-designed inner/outer bending system and stretching test system. The outer bending test induced tensile stress on the film, whereas the inner bending test induced compressive stress. In addition, dynamic fatigue bending tests were carried out using a lab-designed cyclic bending test system at a fixed bending radius of 10 mm and 10 000 bending cycles.

### Fabrication and evaluation of flexible and stretchable TFHs

To demonstrate the feasibility of PTFE/Ag NW as a transparent electrode for flexible and stretchable TFHs, conventional film heaters (50 × 50 mm^2^) with two-terminal side contact were fabricated on the PTFE/Ag NW electrodes. For comparison, bare Ag NWs were also used as a transparent electrode. After wet cleaning of the PTFE/Ag NW electrodes, a 100 nm thick Ag side contact electrode was sputtered on the PTFE/Ag NW electrodes. A DC voltage was supplied by a power supply (OPS 3010, ODA technologies) to the PTFE/Ag NW-based TFHs through the Ag contact electrode at the film edges. The temperature of the TFHs was measured using thermocouples mounted on the surface of the TFHs and an IR thermal imager (A35sc, FLIR) (ESI Fig. S3†).

## Results and discussion

**Fig. 1:**
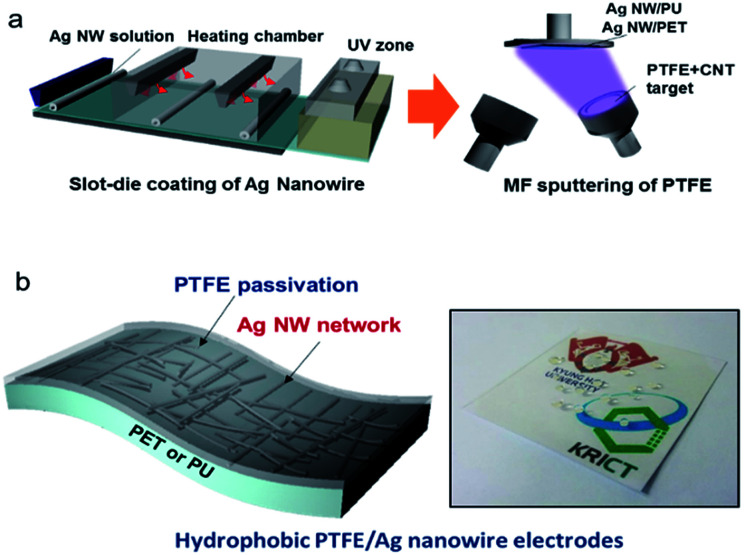
(a) Slot-die coating process of the Ag NW network and MF sputtering process of the hydrophobic PTFE layer on flexible PET and stretchable PU substrates. (b) Schematic of PTFE/Ag NW hybrid electrode on flexible and stretchable substrates. Picture of hydrophobic PTFE/Ag NW electrode on PET substrate; the water droplet on the film indicates the superhydrophobic surface of the PTFE/Ag NW electrode.

**Fig. 2:**
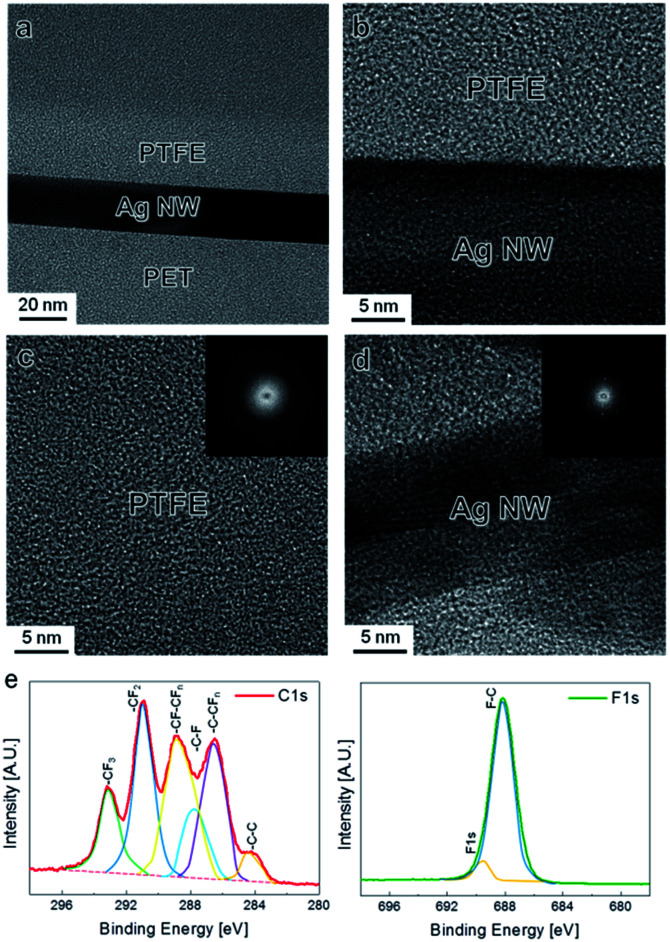
(a) Cross-sectional TEM image obtained from flexible PTFE/Ag NW/PET sample. Enlarged TEM images obtained from (b) interface between PTFE and Ag NWs, (c) PTFE passivation layer and (d) Ag NWs. (e) XPS C 1s and F 1s spectra of MF sputtered PTFE passivation layer on the Ag NW network.

**Fig. 3:**
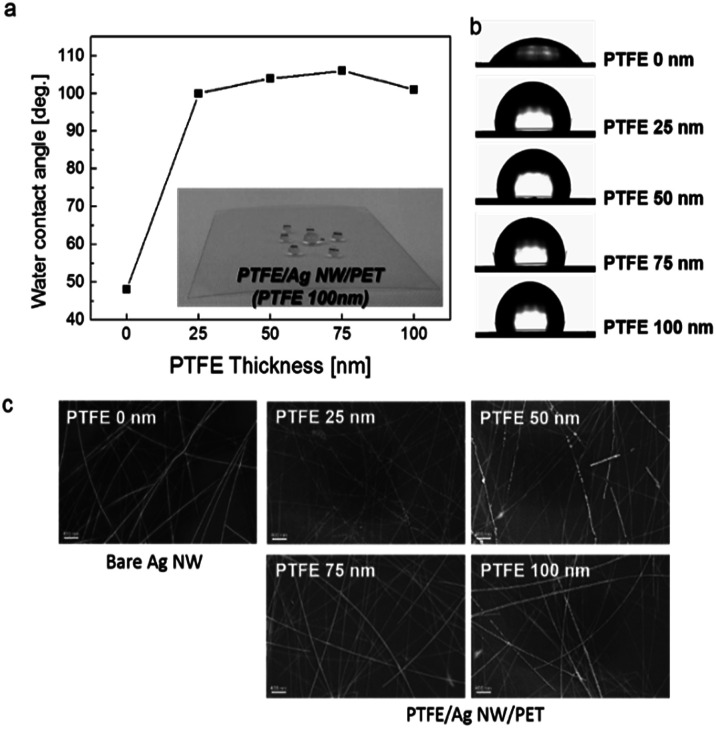
(a) Effect of PTFE thickness on the contact angle of PTFE/Ag NW/PET samples. Inset picture shows a water droplet on the PTFE/Ag NW/PET sample. (b) Shape of a water droplet on a PTFE/Ag NW/PET sample and (c) surface FESEM images of the PTFE-coated Ag NWs with increasing PTFE thickness from 25 nm to 100 nm and the bare Ag network electrode.

To fabricate hydrophobic and flexible/stretchable electrodes on flexible PET and stretchable PU substrates, we used a slot-die coating process for the Ag NW network films and magnetron MF sputtering for the PTFE layer, as shown in Fig. 1(a). The slot-die coating process used for the Ag NWs was described in detail in our previous work.^16^ The Ag NW layer was coated through roll to roll slot die head and exposed the heating zone (120 °C) at a constant speed of 2 m min^−1^. Then, the PTFE layer was deposited as a MF sputtering process under Ar flow 50 sccm, working pressure 7 mtorr and distance between target and substrate 240 mm. In the sputtering process, the heat treatment was not carried out due to degradation PTFE surface and decrease in optical transmittance.^20^ The PTFE/Ag NW hybrid electrode represented by Fig. 1(b), the Ag NW percolating network acted as a flexible or stretchable conduction path for current flow while the PTFE layer acted as a hydrophobic passivation layer to protect the Ag NW network and prevent heat loss from the TFHs. In addition, the PTFE reinforced the Ag NW electrode for when the PET or PU substrate experienced external force by conformally coating the Ag NW network. As illustrated in Fig. 1(b), the PTFE/Ag NW hybrid electrode has a versatile range of properties such as low sheet resistance, high optical transmittance, outstanding flexibility/stability, thermal stability, long lifetime, and superhydrophobic surface (ESI Fig. S1†). It has been reported that the deposition of a PTFE layer on certain surfaces can improve the self-lubrication and water repellence of the surface due to the weak interaction between the C–F superficial molecules.^21–23^ However, PTFE is very difficult to print as a thin film using a solution-based coating process because it cannot be readily dissolved in any solvent. For this reason, a variety of chemical vapor deposition and physical vapor deposition (PVD) techniques have been used to deposit PTFE layers.^24,25^ Although radio frequency (RF) magnetron sputtering has been considered as a simple, large-area coating technique, and is a mature thin film process compared to many PVD techniques, investigations of MF- or DC-sputtered PTFE layers is still lacking due to the absence of a conductive PTFE sputtering target. Due to the insulating properties of white-colored PTFE targets, most sputtered PTFE layers have been deposited by RF magnetron sputtering at a very low deposition rate.^26,27^ Although the advantages of MF magnetron sputtering, such as process stability and high deposition rate, are well known, the characteristics of MF-sputtered PTFE films on Ag NW networks have not yet been reported in detail. Therefore, we used a mixed carbon nanotube (CNT) and PTFE conductive target with a gray color for MF and direct current sputtering, as described in our previous work.^28^ Because the nano-sized PTFE passivation layer prepared by MF sputtering provides not only a hydrophobic surface and mechanical flexibility/stretchability for the Ag NW network electrode but also protection of the Ag NWs, the applications of such Ag NW network electrodes could be diversified in smart windows or wearable electronics. Fig. 2(a) shows a cross-sectional transition electron microscope (TEM) image of a PTFE/Ag NW/PET sample. It is clear that the amorphous PTFE layer conformally covered the crystalline Ag NW with a well-defined interface. In addition, there was no interfacial layer between the Ag NW and the PTFE passivation layers because the MF sputtering process was carried out at room temperature. Fig. 2(b) shows enlarged TEM images of the interface region between the Ag NW and PTFE layers. Although the PTFE layer was directly sputtered on the Ag NWs, there was no damage to the Ag NW by exposure to high-density MF plasma. In addition, the very sharp interface indicates that the PTFE polymer layer effectively passivated the surface of the Ag NW from ambient oxygen or sulfur. The enlarged image of the PTFE passivation layer on Fig. 2(c) shows a typical amorphous structure with short-range order. The diffused fast Fourier transformation (FFT) pattern in the inset of Fig. 2(c) also demonstrates that the MF sputtered PTFE layer had a completely amorphous structure. This amorphous PTFE layer reinforced the Ag NW network by conformally covering the Ag NW and protected the network electrode from sulfur and oxygen in the environment. As we reported in previous works, the slot-die coated Ag NWs have a well-developed crystalline structure with a very smooth (100) side surface plane.^16^ These (100) side surface planes are physically connected to each other, providing a current conduction path in the PTFE/Ag NW electrode. A cross-sectional image of a single Ag NW with a diameter of around 20 nm, Fig. 2(d) showed that the Ag NW was well covered by the MF sputtered PTFE layer. X-ray photoelectron spectroscopy (XPS) analysis was carried out to investigate the chemical composition of the MF sputtered PTFE passivation layer on the Ag NW network. Peak-fitted spectra for the C 1s core and F 1s core level of the PTFE layer are shown in Fig. 2(e). The C 1s and F 1s spectra were deconvoluted into different moieties using Gaussian curve fitting. The spectrum can be satisfactorily fitted by a combination of distinct peaks: the peak at 285.1 eV corresponds to C–C moieties, the peak at 288.2 eV to C–F groups, the peak at 290.8 eV to C–F_2_, the peak at 292.6 eV to C–F_3_, and the peak at 286.7 eV to C–CF_*n*_ groups.^29^ The F 1s spectrum showed a high intensity peak centered at a binding energy of 688.18 eV, which corresponds to F in CF–CF_*n*_ (major peak) and binding energy of around 689.5 eV which corresponds to F in CF_3_ (minor peak). It is well known that the presence of –CF_3_ and –CF_2_ groups on the surface can effectively decrease the surface energy of the film.^30–32^ We also carried out FT-IR analysis in order to verify the presence of PTFE coating on Ag NW networks (ESI Fig. S2†). Therefore, the hydrophobic surface properties of the PTFE/Ag NW electrode can be attributed to the existence of C–F groups on the surface of the MF sputtered PTFE layer. The weak interactions of the C–F groups leads to a self-cleaning and water-resistance surface on the PTFE-coated Ag NW electrodes. Fig. 3(a) shows the effect of the thickness of the MF sputtered PTFE layer on the contact angle of water droplets on the PTFE/Ag NW/PET samples. A contact angle is formed when a water droplet is in thermodynamic equilibrium with a solid such as a film or substrate and exhibits wettability with the surface of the solid. In general, contact angle is used to determine hydrophobic and hydrophilic surfaces.^33^ As the surface energy of a solid surface decreases, the contact angle increases and the wettability decreases. The contact angle *θ* of a liquid droplet on a solid surface is given by the following equation, known as Young's equation.^34^cos *θ* = (*γ*_SV_ − *γ*_SL_)/*γ*_LV_

The components of the equation are the interfacial free energies of the solid–vapor (*γ*_SV_), solid–liquid (*γ*_SL_), and liquid–vapor (*γ*_LV_) interfaces. The bare Ag NW film without a PTFE layer had a low contact angle of 48°, as shown in Fig. 3(a). However, after the PTFE passivation layer was coated on the Ag NW network electrodes, the contact angle abruptly increased. Regardless of PTFE thickness, all PTFE/Ag NW electrodes showed high contact angles of greater than 100 degree due to the low surface energy of the MF sputtered PTFE layer. At a PTFE thickness of 75 nm, the PTFE/Ag NW electrode demonstrated the highest contact angle of 106 degree, which is sufficient for fabrication of self-cleaning smart windows.^35–38^ The inset picture shows the water droplet formed on the hydrophobic PTFE (75 nm)/Ag NW electrode before the fabrication of TFHs. Fig. 3(b) shows the water droplet shape on the PTFE/Ag NW/PET sample as a function of the PTFE thickness. It is clearly shown that the existence of the MF sputtered PTFE layer on the Ag NW network led to an abrupt increase in contact angle, indicating the superhydrophobic surface of the PTFE/Ag NW electrode. The surface field emission scanning electron microscopy (FESEM) images of the PTFE layer coated on the Ag NW network, Fig. 3(c) show that the MF sputtered PTFE layer conformally covered the Ag NW without surface defects such as cracks, pinholes, or delamination. In addition, the connectivity of the Ag NW network was not affected by the MF sputtered PTFE passivation layer because the MF sputtering process was carried out at room temperature without heating the substrate. Furthermore, we employed an MF power source for the sputtering of PTFE, so the surface heating by plasma irradiation was fairly small compared to that of conventional DC sputtering.^39^

**Fig. 4:**
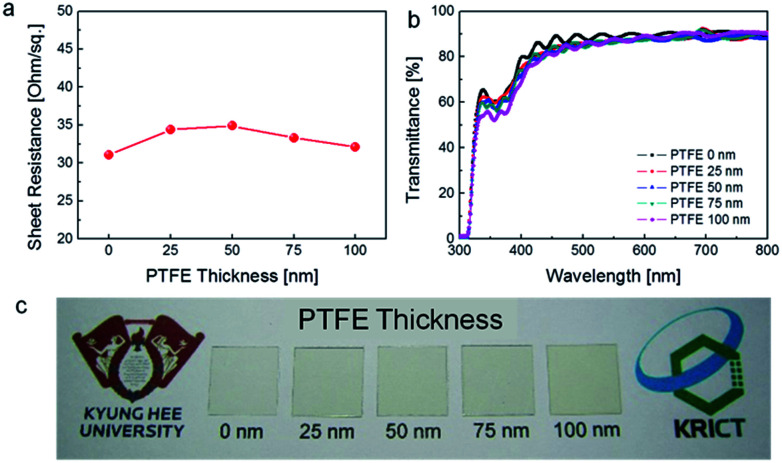
(a) Sheet resistance and (b) optical transmittance of the PTFE/Ag NW electrodes on flexible PET substrates with increasing PTFE thickness from 0 to 100 nm. (c) Picture showing the transparency and color of the PTFE/Ag NW electrodes on PET substrates.

Fig. 4(a) shows the sheet resistance of the PTFE/Ag NW electrode fabricated on PET substrates as a function of PTFE thickness. Due to the insulating properties of the PTFE passivation layer, all PTFE/Ag NW electrodes had a similar sheet resistance to the bare Ag NW electrode. The average of sheet resistance is 31 ohm per square. It was noteworthy that the existence of the PTFE passivation layer did not affect the electrical properties of the Ag NW network electrode because the connectivity of the Ag NW was not affected by the PTFE sputtering and thickness, as expected based on the FESEM images shown in Fig. 3(c). Fig. 4(b) shows the optical transmittance of the PTFE/Ag NW and bare Ag NW electrodes fabricated on PET substrates. With increasing PTFE thickness, the optical transmittance of the PTFE/Ag NW electrodes slightly decreased. Although the PTFE/Ag NW electrode has a slightly lower optical transmittance than the bare Ag NW electrode, all PTFE/Ag NW samples showed an optical transmittance at 550 nm wavelength of above 85%, which is acceptable for the fabrication of transparent TFHs. However, the PTFE-coated Ag NW electrodes had a slightly yellowish color, as shown in Fig. 4(c) which indicated PTFE/Ag NW electrodes depending on PTFE thickness.

**Fig. 5:**
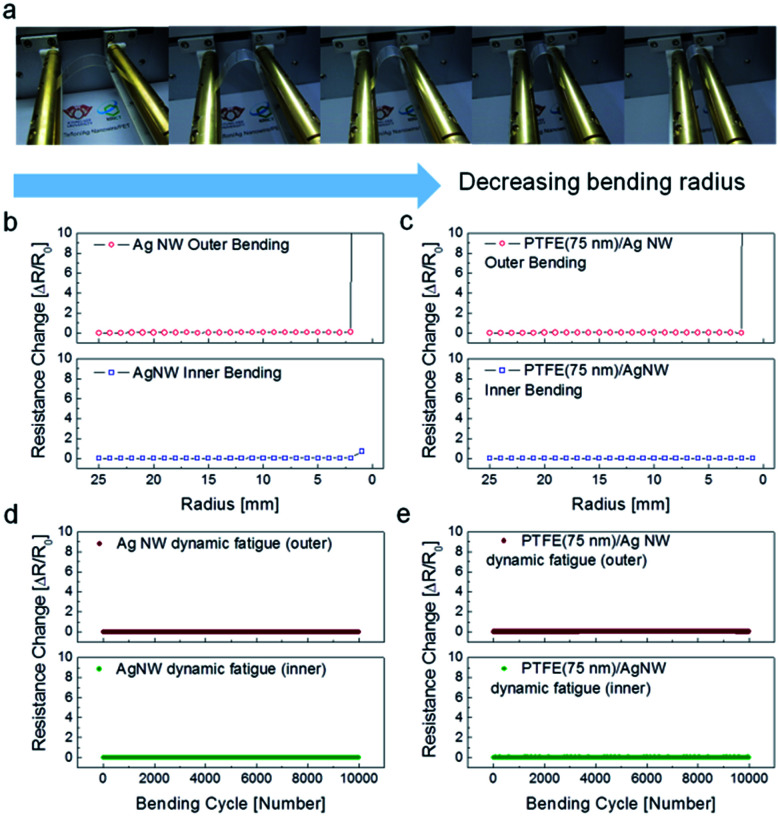
(a) Pictures of operated bending tester with decreasing bending radius. (b) Outer and inner bending test results for the bare Ag NWs. (c) Outer and inner bending test results for the optimized PTFE/Ag NW electrode. Dynamic fatigue test results for (d) Ag NW electrode and (e) PTFE/Ag NW electrode with increasing number of bending cycles. Upper panels show an outer bending step for the dynamic fatigue test.

**Fig. 6:**
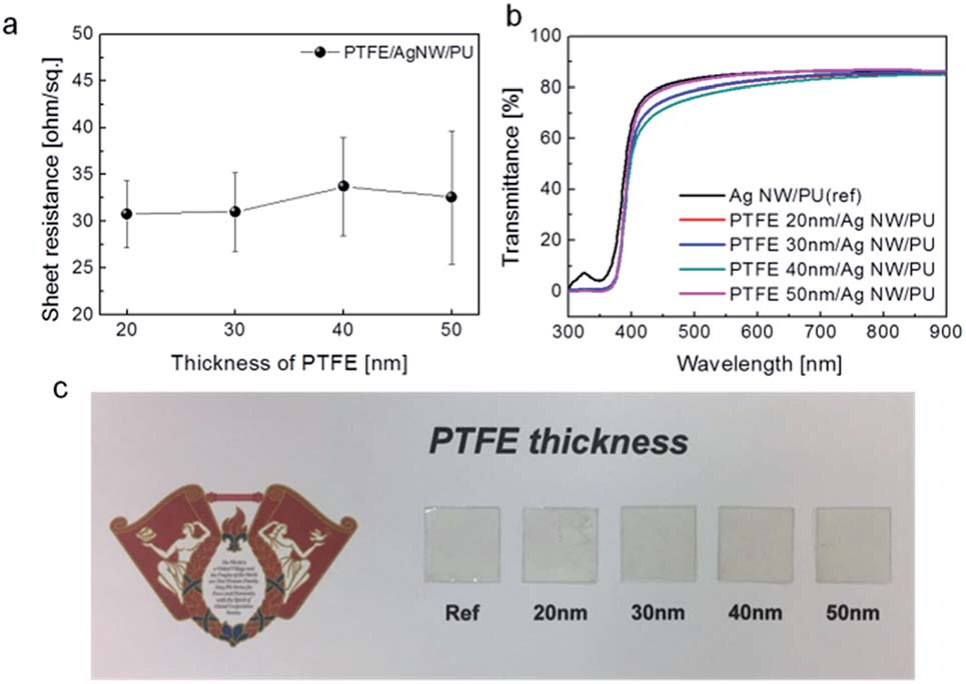
(a) Sheet resistance and (b) optical transmittance of the PTFE/Ag NW electrodes on stretchable PU substrates as a function of PTFE thickness. (c) Picture comparing the transparency and color of the Ag NW/PU and PTFE/Ag NW/PU samples with increasing PTFE thickness.

To investigate the flexibility of the PTFE/Ag NW electrodes on PET substrates, resistance change was measured as a function of bending radius, as shown in Fig. 5(a), using a lab-designed inner/outer bending test system. Fig. 5(b) and (c) compare the inner bending and outer bending test results of the bare Ag NW electrode and the PTFE/Ag NW electrode with a 75 nm thick PTFE layer. The resistance change was plotted as a function of outer and inner bending radius change. The change in resistance of the electrodes was calculated as Δ*R* = (*R* − *R*_0_)/*R*_0_. Here, *R*_0_ is the initial resistance value and *R* is the resistance after substrate bending. It is noteworthy that the inner and outer bending test results were very similar for the PTFE/Ag NW electrode and the bare Ag NW electrode. Both the Ag NW and the PTFE/Ag NW electrode showed similar critical bending radii of 3 mm. Compared to the outer bending test, the inner bending test showed a lower resistance change due to the overlapping of the cracked Ag NW or PTFE layer.^40^Fig. 5(c) and (d) show the resistance change during dynamic outer and inner bending tests for the bare Ag NW and PTFE/Ag NW electrodes at a fixed bending radius of 10 mm. As expected from the inner and outer bending test results, both samples showed a constant resistance change for 10 000 cycles under repeated compressive and tensile stress, regardless of the bending mode. Therefore, it was concluded that the existence of the MF sputtered PTFE passivation layer did not affect the electrical, optical, or mechanical properties of the Ag NW electrode. However, due to the stretchability limit of the PET substrate (∼3%), the PTFE/Ag NW/PET electrode could only be used in flexible TFHs.^41^ To apply the PTFE/Ag NW electrode in stretchable electronics, we also prepared PTFE/Ag NW electrodes on the stretchable PU substrate. Fig. 6(a) shows the sheet resistance of PTFE/Ag NW electrodes fabricated on stretchable PU substrates for applications in stretchable and transparent TFHs. Prior to PTFE sputtering, the Ag NW network was coated on the PU substrate under the same slot-die coating conditions used in coating the Ag NW on the PET substrate. Because the stretchable PU substrate was severely affected by plasma irradiation during PTFE sputtering, we prepared a thinner PTFE passivation layer in the Ag NW/PU samples than that used in the Ag NW/PET samples. As expected from Fig. 4(a), the sputtered PTFE layer on the PU substrate also did not affect the sheet resistance of the Ag NW network electrode (30 ± 3.7 ohm per square). Therefore, like the PTFE/Ag NW/PET samples, all PTFE/Ag NW electrodes on PU substrates showed similar sheet resistance, regardless of the PTFE thickness. Fig. 6(b) shows the optical transmittance of the PTFE/Ag NW electrodes prepared on stretchable PU substrates as a function of PTFE thickness from 0 nm to 50 nm. The bare Ag NW on a PU substrate showed a high optical transmittance of 84.91% at a 550 nm wavelength. The sputtering of the PTFE layer on the Ag NW/PU sample led to a slight decrease in optical transmittance. The stretchable PTFE/Ag NW/PU samples demonstrated lower optical transmittance than the flexible PTFE/Ag NW/PET samples despite the thinner PTFE layer due to the low optical transparency (90.02%) of the PU substrate. As the PTFE thickness increased, the PTFE/Ag NW/PU sample showed a decrease in optical transmittance. Fig. 6(c) shows a picture of PTFE/Ag NW/PU samples as a function of PTFE thickness. Due to the high optical transparency of PTFE and Ag NW, the hybrid electrodes also have high transparency; however, they also have a slightly yellowish color.

**Fig. 7:**
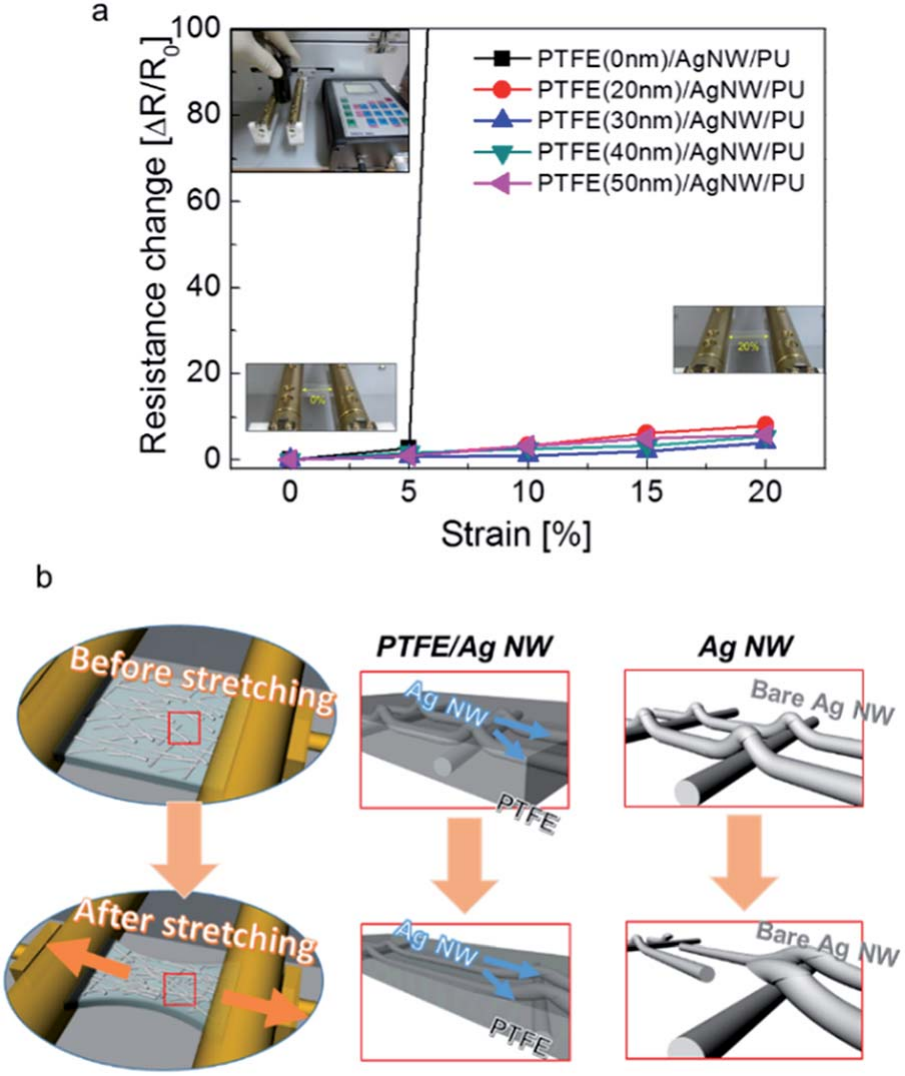
(a) Relative resistance change of PTFE/Ag NW electrodes with increasing strain as a function of the PTFE thickness. (b) Reinforcement mechanism of PTFE-coated Ag NW hybrid electrode compared to the bare Ag NW electrode when the substrate was stretched.

Fig. 7(a) demonstrates the stretching test results for the PTFE/Ag NW/PU sample using a specially designed electrode stretching test system. A one-dimensional strain was gradually applied to the PTFE/Ag NW/PU samples from 0% to 20%. The change in resistance of the electrode during substrate stretching can be expressed as Δ*R* = (*R* − *R*_0_)/*R*_0_, where *R*_0_ is the initial resistance and *R* is the resistance measured under stretched conditions. In the case of the bare Ag NW electrode coated on the PU substrate, there was an abrupt resistance change within 10% strain due to the delamination of the Ag NW or disconnection in the Ag NW network.^42^ However, the PTFE/Ag NW/PU samples showed fairly constant resistance until a strain of 20%. The sputtered PTFE layer improved the adhesion of the Ag NW network by covering the Ag NWs, and reinforced the PTFE/Ag NW hybrid electrode during stretching. The outstanding stretchability of the PTFE/Ag NW/PU indicates that PTFE/Ag NW is a promising electrode for stretchable TFHs. Fig. 7(b) is a schematic of the stretching mechanism of the PTFE/Ag NW/PU sample during the stretching test. In the case of the bare Ag NW network, the physical contacts between Ag NW were disconnected when the substrate was stretched. In addition, the weak bond of the Ag NWs with the PU substrate led to delamination of the Ag NW network. Furthermore, the short Ag NWs were disconnected when the sample was stretched, as illustrated in Fig. 7(b). However, the PTFE/Ag NW electrode showed outstanding stretchability due to the existence of the polymer PTFE layer conformally covering the Ag NWs. During stretching of the sample, the stretched PTFE layer reinforced the Ag NWs and improved the adhesion of the Ag NW to the PU substrate by covering the Ag NW network. Because the PTFE layer strongly held the Ag NWs during stretching, the PTFE/Ag NW hybrid electrode showed superior stretchability to the bare Ag NW electrode.

**Fig. 8:**
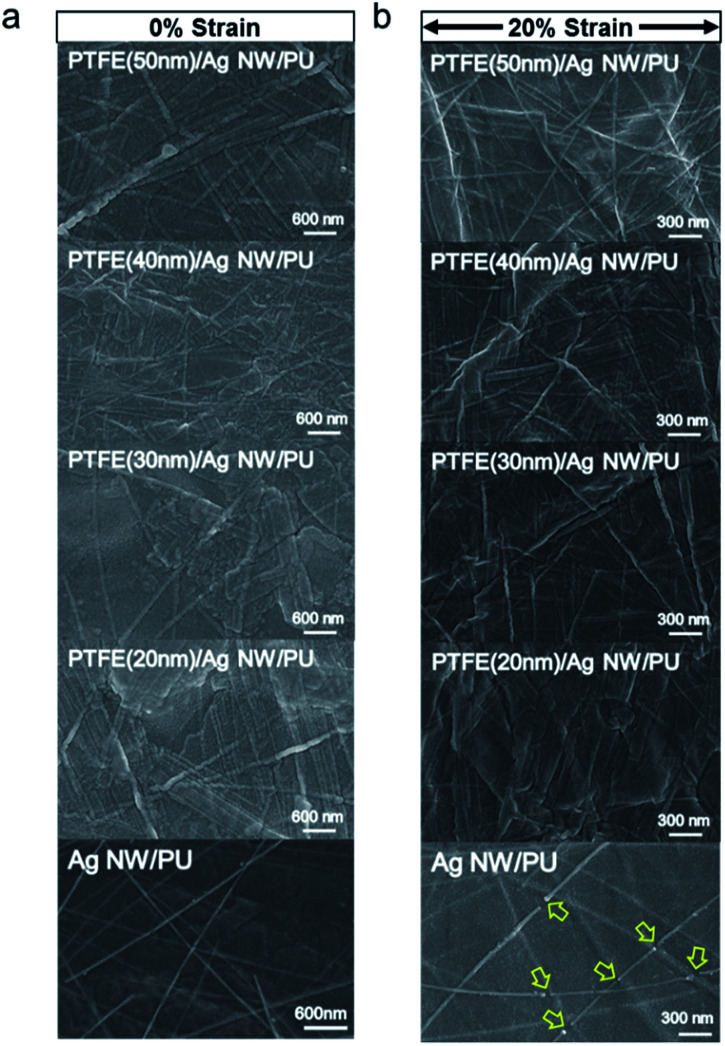
Surface FESEM images of the PTFE/Ag NW and bare Ag NW electrodes on PU substrates (a) before stretching and (b) after 20% stretching as a function of PTFE thickness.

**Fig. 9:**
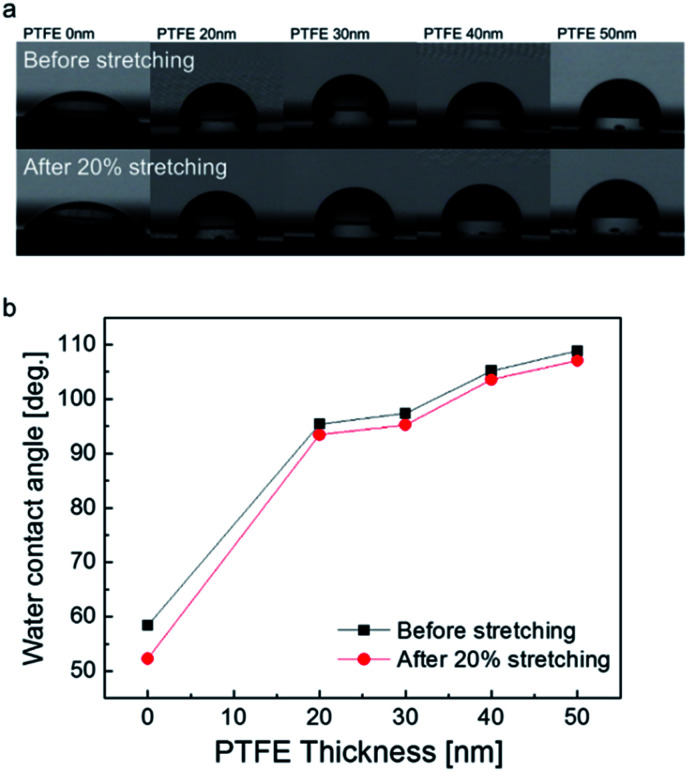
(a) Water droplet on a PTFE/Ag NW electrode on the PU substrate before and after 20% stretching. (b) Correlation between PTFE thickness and contact angle before and after stretching of 20%.

Fig. 8(a) and (b) compare the surface FESEM images of the bare Ag NW/PU and PTFE/Ag NW/PU samples before and after 20% stretching as a function of PTFE thickness. The bare Ag NW/PU sample showed a well-connected Ag NW network before stretching. However, after 20% stretching, some regions of the Ag NWs were disconnected, as indicated by the arrow. Due to the disconnected or delaminated Ag NWs, the resistance of the Ag NW network gradually increased with increasing strain, even after only 5%. The short Ag NWs were physically disconnected when the PU substrate was stretched. Surface FESEM images of the PTFE/Ag NW/PU samples before stretching showed typical amorphous surface features without surface defects. After 20% stretching, all PTFE/Ag NW/PU surface FESEM images appeared identical to those of the as-deposited samples. Due to the outstanding stretchability of the sputtered PTFE passivation layer, the PTFE-coated Ag NWs showed better stretchability than the bare Ag NW network. Regardless of the PTFE thickness, all PTFE/Ag NW/PU samples had identical surface images before and after stretching, which is consistent with the resistance change results in Fig. 7(a). This indicates that the PTFE/Ag NW electrode prepared on a PU substrate is a promising stretchable electrode for stretchable and self-cleaning TFHs. Fig. 9(a) and (b) show water droplet images and contact angles on the PTFE/Ag NW/PU samples as a function of the sputtered PTFE layer thickness. Like the Ag NW electrode coated on PET substrate shown in Fig. 3(b), the bare Ag NW coated on PU substrate showed a low contact angle of 58 degree due to the hydrophilic properties of metallic Ag NWs. The PTFE coated on the Ag NW electrodes, however, showed increased contact angle due to the hydrophobic surface properties of the PTFE passivation layers. With increasing PTFE thickness, the PTFE/Ag NW electrode showed a linearly increasing contact angles from 58 degree to 108 degree, as shown in Fig. 9(b). At a PTFE thickness of 50 nm, the PTFE/Ag NW electrode had a contact angle of 108 degree, which is acceptable for the fabrication of stretchable and self-cleaning TFHs. It is noteworthy that even after 20% stretching, the PTFE/Ag NW electrode showed similar contact angles to that of the as-deposited samples due to the hydrophobic surface of the stretched PTFE layer.

**Fig. 10:**
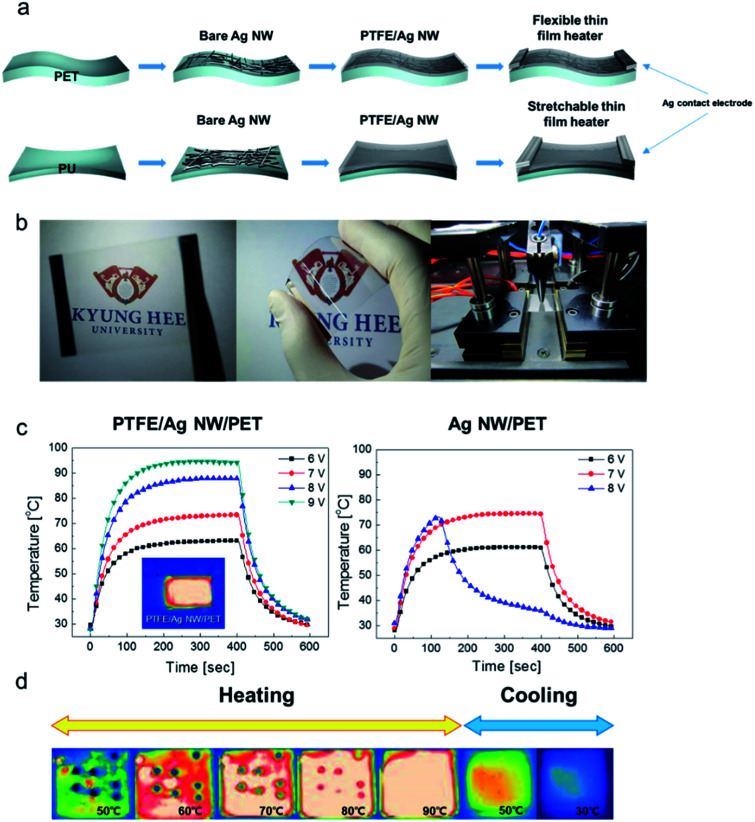
(a) Schematic of the fabrication process for flexible and stretchable TFHs using PTFE/Ag NW/PET and PTFE/Ag NW/PU, respectively. (b) Pictures of flexible TFHs fabricated on PTFE/Ag NW/PET substrates, and temperature measurements. (c) Temperature profiles of the flexible TFHs fabricated on PTFE/Ag NW/PET and Ag NW/PET samples as a function of input voltage. (d) IR images of the hydrophobic TFHs fabricated on PTFE/Ag NW/PET substrate. Water droplets on the hydrophobic TFHs disappeared with increasing surface temperature.

To investigate the feasibility of flexible PTFE/Ag NW/PET and stretchable PTFE/Ag NW/PU samples as flexible and stretchable electrodes, we fabricated flexible and stretchable transparent TFHs with superhydrophobic surfaces. Fig. 10(a) demonstrates the fabrication process for flexible and stretchable transparent TFHs using PTFE/Ag NW/PET and PTFE/Ag/PU, respectively. A DC voltage was applied to the stretchable and flexible TFHs by a DC power supply through the sputtered Ag electrodes at the sample edge, as shown in Fig. 10(a), and the temperature profile was measured by a thermocouple placed on the surface of the electrode which is represented in Fig. 10(c). Furthermore, infrared (IR) thermal images were obtained using an IR thermometer. Fig. 10(b) shows pictures of the flexible and hydrophobic TFHs fabricated on PTFE/Ag NW/PET sample as well as the surface temperature measurements. Fig. 10(c) compares the temperature profiles of the flexible and hydrophobic TFHs fabricated on PTFE/Ag NW/PET and bare Ag NW/PET samples, plotted with respect to input voltage. When DC input voltage was supplied to the PTFE/Ag NW electrode-based TFHs, the temperature of the flexible TFHs gradually increased and reached a maximum. As the input voltage increased, the saturation temperature of the flexible TFHs increased, as shown in all of the temperature profiles. When the DC voltage supplied was 6 V, the flexible and hydrophobic TFH with PTFE/Ag NW electrode reached 63.1 °C. A further increase in the applied DC voltage to 9 V led to an increase in the saturation temperature to 94.2 °C. However, TFHs fabricated on a bare Ag NW electrode with sheet resistance of 31.1 ohm per square reached only 74.6 °C under an applied DC voltage of 7 V. When a DC voltage of 8 V was applied to the bare Ag NW/PET electrode, it did not reach a saturation temperature and the temperature of the TFH abruptly decreased due to the disconnection of the Ag NW electrode, as shown in Fig. 10(c). The more efficient transduction of electrical energy in the flexible TFHs with PTFE/Ag NW electrode was attributed to the low sheet resistance of the transparent electrode and the passivation effect of the PTFE layer, which has high *T*_g_.^43–47^Fig. 10(d) shows IR images of the flexible and hydrophobic TFH with a saturation temperature of 94.2 °C. Due to the hydrophobic surface of the sputtered PTFE passivation layer, several water droplets were formed on the surface of the TFHs. When DC input voltage of 9 V was supplied to the flexible TFHs, a saturation temperature of 94.2 °C was achieved when Joule heating and convection reached a dynamic balance. The water droplets disappeared almost immediately due to the high surface temperature of the flexible and hydrophobic TFHs. The rapid disappearance of the water droplets indicates that the PTFE/Ag NW/PET sample is a promising flexible and self-cleaning TFH that could be attached to curved surfaces or curved windows of automobiles.

**Fig. 11:**
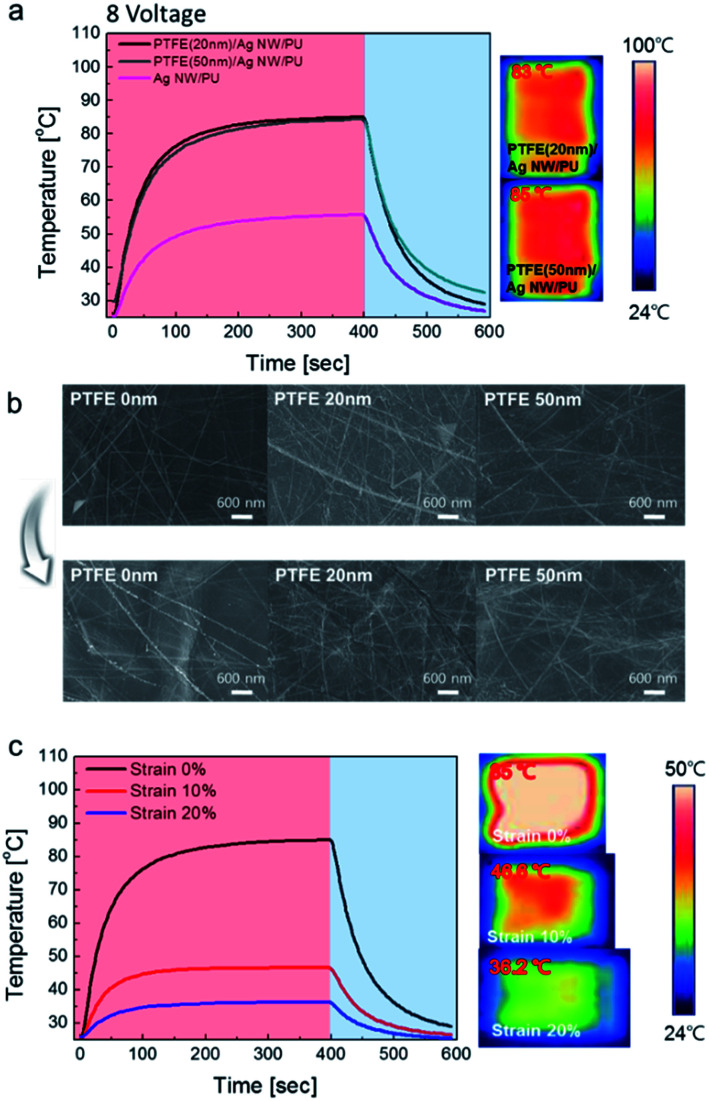
(a) Temperature profiles of the PTFE/Ag NW based TFHs and IR images. (b) Surface FESEM images of bare Ag NW and PTFE/Ag NW electrodes before and after DC power application. (c) Temperature profile of stretchable TFHs with PTFE/Ag NW electrodes as a function of strain while operating at 8 V of input voltage.

Fig. 11(a) shows temperature profiles of the PTFE/Ag NW-based stretchable and hydrophobic TFHs, plotted with respect to PTFE layer thickness. When DC voltage was supplied to the PTFE/Ag NW or Ag NW based TFHs, the temperature of both stretchable TFHs gradually increased and reached a maximum. However, it was clear that the saturation temperature of the PTFE/Ag NW-based TFHs (85 °C) was much higher than that of the bare Ag NW based TFH (55.7 °C) at same input voltage of 8 V. Due to the thermoplastic properties of the PU substrate, we cannot increase the temperature above 85 °C.^48^ The high saturation temperature of the PTFE/Ag NW based TFHs could be attributed to the stability of the PTFE/Ag NW electrodes, as shown in Fig. 11(b). Although the bare Ag NW on PU substrate had a similar resistance to the PTFE/Ag NW electrode on the same substrate, there was a lot of heat loss in the bare Ag NW electrode without a passivation layer. However, the PTFE passivation layer on the Ag NW electrode preserved the heat produced by the Ag NW network and maintained a higher saturation temperature, as well as providing a hydrophobic surface. We compared surface FESEM images of the stretchable TFHs with bare Ag NW and PTFE/Ag NW electrodes before and after sample heating. In the case of bare Ag NW based stretchable TFHs, there were disconnected regions in the Ag NW network after heating, like in the Ag NW/PET sample. However, for the PTFE/Ag NW based TFHs, the surface FESEM images of the PTFE layer after heating was identical to that of the as-deposited sample. This demonstrates the thermal stability of the MF sputtered PTFE passivation layer. Fig. 11(c) shows temperature profiles of the stretchable TFH with the PTFE (50 nm)/Ag NW electrode. The temperature of the stretchable thin film heaters was plotted as a function of strain from 0% to 20% at a constant input voltage of 8 V. In the case of the stretchable TFH on the PTFE/Ag NW electrode without strain, the temperature reached 85 °C at a constant voltage of 8 V. However, with increasing strain from 0 to 20%, the maximum temperature gradually decreased to 36.2 °C due to the increase in heat-conducting paths caused by stretching of the Ag NW network at a constant input power. In addition, a slight increase in the sheet resistance by stretching led to a decrease in the maximum saturation temperature. This is because the maximum temperature of stretchable thin film heaters is closely related to the electrode resistance.
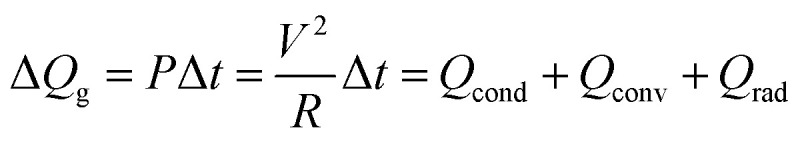


As shown in the equation of Joule's law, we can correlate the sheet resistance of transparent electrodes for thin film heaters and the generated temperature.^49,50^ The power (*P*) applied to the thin film heaters during the heating time (Δ*t*) generates heat (Δ*Q*_g_), where *V* is the input DC voltage between the contact electrodes, *R* is the resistance of the thin film heaters, *Q*_cond_ is the heat loss due to conduction in the substrate, *Q*_conv_ is the heat loss due to convection in air, and *Q*_rad_ is the heat loss due to radiation. Based on the equation above, it is apparent that the maximum temperature of the thin film heaters decreases with increasing resistance (*R*) under a constant input DC voltage (*V*). The decrease in maximum temperature with increasing strain for the PU substrate was therefore attributed to the increase in sheet resistance with increasing strain. However, even at a high strain of 20%, we observed successful operation of the thin film heaters, indicating that Ag NWs on a PU substrate can be used as high performance stretchable electrodes.

**Fig. 12:**
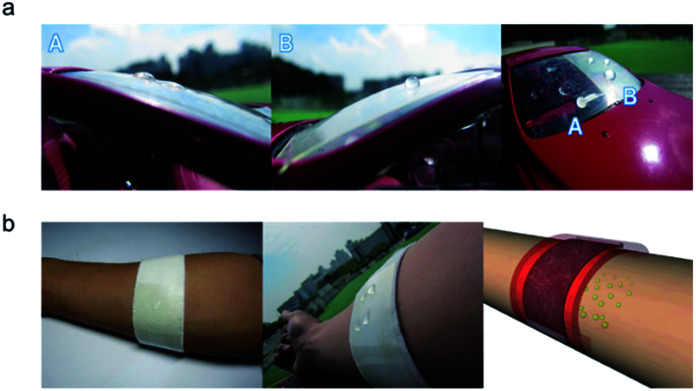
Promising applications of PTFE/Ag NW/PET and PTFE/Ag NW/PU samples. (a) Transparent TFHs attached to the curved window of an automobile can be used as a defogging and deicing system. PTFE/Ag NW/PET based flexible TFH sample was attached to the self-cleaning window of the automobile. The A region is the bare Ag NW electrode and the B region is the PTFE/Ag NW electrode. (b) The stretchable TFHs attached to medical patches enhanced drug diffusion through the skin. The hydrophobic properties of the PTFE/Ag NW/PU based stretchable TFHs led to a waterproof effect.

Fig. 12 demonstrates promising applications of flexible, stretchable and hydrophobic TFHs. First, the PTFE/Ag NW electrodes prepared on flexible PET substrates could be employed as a defogging/deicing window for automobiles, as shown in Fig. 12(a). Because most automobile windows have a curved surface, the flexible and hydrophobic TFHs are appropriate TFHs for self-cleaning TFHs. The hydrophobic surface of the PTFE layer on the window makes the surface of window very clean from dewdrops or raindrops. Based on the comparison in Fig. 12(a), the raindrops on the bare Ag NW electrode (Region A) make water stains on the automobile window due to their small contact angles. However, the raindrops on the PTFE/Ag NW electrode (Region B) keep the surface of the window clean because the raindrops have a high contact angle and are thus thrown away when the automobile moves. Another promising application of the PTFE/Ag NW based TFH is a wearable heating pain relief patch, as shown in Fig. 12(b). The TFH equipped in the patch could help the diffusion of the drug around the injured area through the heating of human skin. To apply the TFH in a medical patch, the stretchability of the TFH is very important. Therefore, the PTFE/Ag NW electrode based TFH fabricated on PU substrates is an appropriate TFH for attachable medical patches on the human body. In addition, the stretchable TFH with hydrophobic surfaces is beneficial because the surface of the patch was not affected by water droplets or raindrops. Therefore, a cost-effective and self-cleaning transparent TFH with flexibility and stretchability could be realized by PTFE/Ag NW electrodes on either PET or PU substrates.

## Conclusion

In summary, we investigated the electrical, optical, morphological, and mechanical properties of PTFE-coated Ag NW electrodes fabricated on PET and PU substrates, respectively, to use as hydrophobic flexible/stretchable, transparent electrodes. Using a mixed CNT and PTFE target, we successfully sputtered a PTFE passivation layer on the slot-die coated Ag NW electrode without changing the hydrophobic surface properties of the PTFE. The hydrophobic surface of the PTFE/Ag NW electrodes led to water-repelling and self-cleaning Ag NW electrodes which is beneficial for transparent TFH-based smart windows. To optimize the thickness of the PTFE layer on the Ag NW electrode, we investigated the effect of PTFE thickness on the electrical properties, optical properties, and wetting angle of the PTFE-coated Ag NW electrodes. In addition, the mechanical flexibility and stretchability of the hydrophobic PTFE/Ag NW electrodes coated on polyethylene terephthalate (PET) and polyurethane (PU) substrates were investigated in detail. Based on the results of bending and stretching tests, we demonstrated the superior mechanical properties of the PTFE/Ag NW electrode compared to conventional ITO electrodes. Furthermore, we demonstrated the feasibility of the PTFE/Ag NW electrode coated on a PU substrate as a promising electrode for stretchable and self-cleaning TFHs. The effective heat generation of the PTFE/Ag NW electrodes indicates the possibility for energy-efficient multi-functional PTFE/Ag NW TFHs attached to automobile windows, smart windows, and bio-patches.

## Conflicts of interest

There are no conflicts to declare.

## Supplementary Material

RA-008-C8RA00880A-s001
