# Supplementary material for: Hydrophobic and stretchable Ag nanowire network electrode passivated by a sputtered PTFE layer for self-cleaning transparent thin film heaters
Source: RSC Adv. 2018 May 22;8(33):18508–18. doi: 10.1039/c8ra00880a (PMC9080517; doi:10.1039/c8ra00880a)
Supplement: RA-008-C8RA00880A-s001 [file RA-008-C8RA00880A-s001.pdf]

Electronic Supplementary Material (ESI) for RSC Advances.
This journal is © The Royal Society of Chemistry 2018

Supporting Information

Hydrophobic and stretchable Ag nanowire network electrodes passivated by sputtered PTFE layer for self-cleaning transparent thin film heaters

Sang-Mok Lee[a], Sung Hyun Kim[b], Jae Heung Lee[b], Sang-Jin Lee[b] and Han-Ki Kim[c]*

[a]Kyung Hee University, Department of Advanced Materials Engineering for Information and Electronics, 1 Seocheon-dong, Yongin-si, Gyeonggi-do 446-701, Republic of Korea

[b]Chemical Materials Solutions center, Korea Research Institute of Chemical Technology, 141 Gajeongro, Yuseong, Daejeon, 305-600, Republic of Korea

[c]School of Advanced Materials Science & Engineering, Sungkyunkwan University, 2066 Seobu-ro, Jangan-gu, Suwon, Gyeonggi-do, 440-746, Republic of Korea

E-mail:hankikim@skku.edu

Water repellent properties of sputtered PTFE films. We compared the hydrophobic surface properties of PTFE/Ag NW and bare Ag NW electrodes as shown in Figure S1**.** Firstly, we dropped water on the flat PTFE/Ag NW and bare Ag NW films using a typical speed (Figure S1a). The water droplet on the tilted samples clearly showed the effect of the PTFE passivation layer, as shown in Figure S1a. The water droplets on the PTFE/Ag NW electrode flowed down, while the water droplets on the bare Ag NW remained on the surface due to the hydrophilic surface of the Ag NWs. In figure S1b, as soon as we dropped water on the PTFE/Ag NW films, we found water droplets with high contact angle due to the low surface energy of the PTFE films. However, the water droplet on the bare Ag NW spread and made a water stain on the surface of Ag NW electrode.

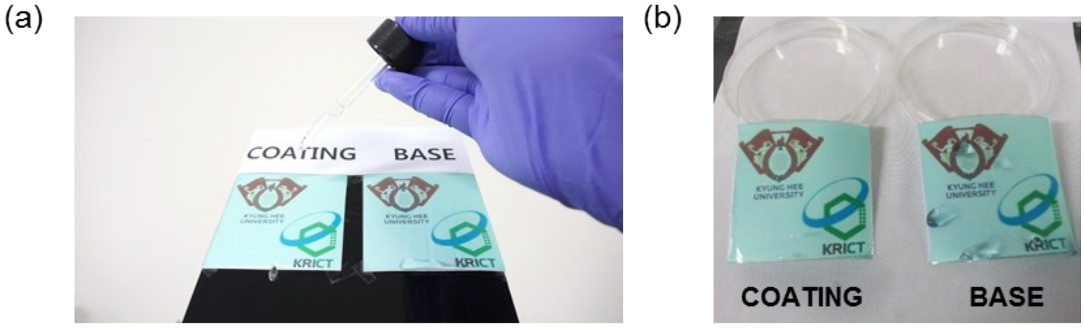

Figure S1. (a) PTFE-coated Ag NW thin film and bare Ag NW thin film layers were prepared to identify the hydrophobic properties. (b) The result of the surface states of the two thin film layers after the water drop.

Analysis of PTFE layer by means of FTIR reflectance absorption spectra. To examine the presence of PTFE layer on Ag nanowire electrode, we analyze the PTFE layer by using FT-IR reflectance absorption spectra. As shown in Figure S2, the peaks observed in the present work are assigned as follows: the three main peak of CF_2 symmetric stretching at 1220, CF_2 asymmetric strethcing at 1153, CF_3 symmetric stretching at 1058 cm^{-1}. The observed minor peaks at 1711 cm^{-1} is attributed to C=C stretching vibration and betwwen 500 and 1000 cm^{-1} are CF_2 wagging, deformation and rocking mode and CF_3 defromation mode [1].

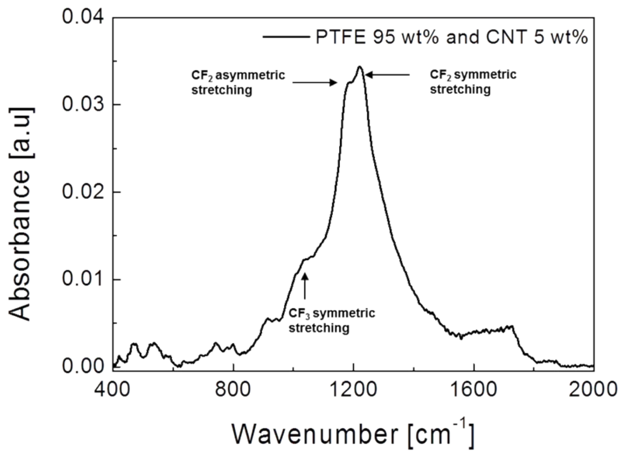

Figure S2. FT-IR reflectance absorption spectrum of MF sputtering PTFE 95 wt% and CNT 5 wt% thin film on Ag nanowire.

Analysis of TFH fabrication based on PTFE/Ag NW electrodes. To demonstrate the effect of the PTFE passivation layer on the Ag NW electrodes, we fabricated thin film heaters and compared the performance of the TFHs based on PTFE/Ag NW electrode and bare Ag NW electrodes. Two terminal side Ag metal contacts were sputtered onto the edge of the PTFE-coated Ag NW and bare Ag NW electrodes to apply power to the TFHs. As shown in Figure S3, a DC voltage was supplied by a power supply (OPS 3010, ODA Technologies) to the PTFE-coated Ag NW–based TFHs through an Ag contact electrode at the film edge, which was clamped. In addition, the temperature of the TFHs was observed using an IR thermal imager (A35sd, FLIR) on the surface of the TFHs, as shown in Figure S2.

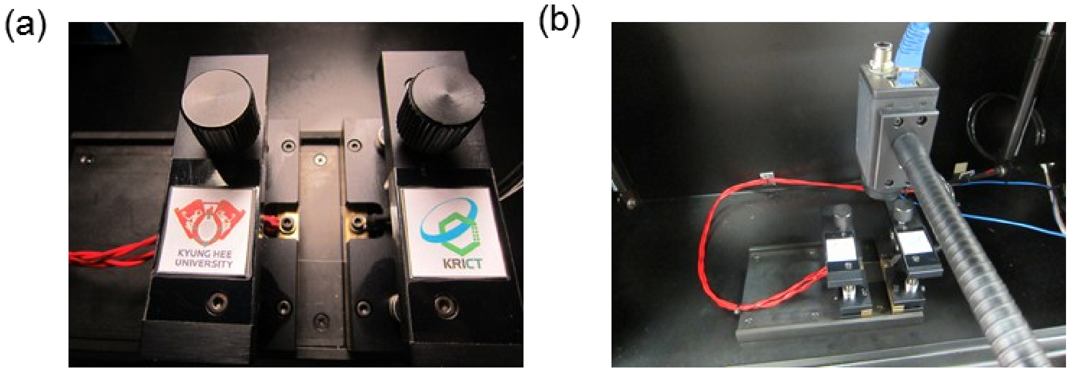

Figure S3. (a) Picture of the PTFE/Ag NW based TFHs connected to DC power in the temperature measurement system. (b) IR thermal imagers for mapping the temperature of the TFHs and temperature uniformity.

REFERENCES

[1] S.K. Bhullar, A. Bedeloglu, M.B.G. Jun, Int. J. Adv. Sci. Eng. 2014, 1, 8
